# An enhancer peptide for membrane-disrupting antimicrobial peptides

**DOI:** 10.1186/1471-2180-10-46

**Published:** 2010-02-15

**Authors:** Satoshi Ueno, Kohtaro Kusaka, Yasushi Tamada, Hong Zhang, Masaomi Minaba, Yusuke Kato

**Affiliations:** 1 Division of Insect Sciences, National Institute of Agrobiological Sciences, Oowashi 1-2, Tsukuba, Ibaraki 305-8634, Japan; 2 Graduate School of Life and Environmental Sciences, University of Tsukuba, Tennoudai 1-1-1, Tsukuba, Ibaraki 305-8572, Japan

## Abstract

**Background:**

NP4P is a synthetic peptide derived from a natural, non-antimicrobial peptide fragment (pro-region of nematode cecropin P4) by substitution of all acidic amino acid residues with amides (i.e., Glu → Gln, and Asp → Asn).

**Results:**

In the presence of NP4P, some membrane-disrupting antimicrobial peptides (ASABF-α, polymyxin B, and nisin) killed microbes at lower concentration (e.g., 10 times lower minimum bactericidal concentration for ASABF-α against *Staphylococcus aureus*), whereas NP4P itself was not bactericidal and did not interfere with bacterial growth at ≤ 300 μg/mL. In contrast, the activities of antimicrobial agents with a distinct mode of action (indolicidin, ampicillin, kanamycin, and enrofloxacin) were unaffected. Although the membrane-disrupting activity of NP4P was slight or undetectable, ASABF-α permeabilized *S. aureus *membranes with enhanced efficacy in the presence of NP4P.

**Conclusions:**

NP4P selectively enhanced the bactericidal activities of membrane-disrupting antimicrobial peptides by increasing the efficacy of membrane disruption against the cytoplasmic membrane.

## Background

Antimicrobial peptides (AMPs) are peptides that are selectively toxic against microbes. To date, more than 800 AMPs have been discovered in various organisms including vertebrates, invertebrates, plants, protozoans, and microbes. The structures of AMPs are extremely diverse. They are categorized into distinct structural groups such as amphipathic α-helical peptides, and β-sheet peptides stabilized by intramolecular disulfide bridges [1]. Several AMPs are already in practical use. For instance, nisin is a widely used food-preservative in more than 50 countries including the United States of America, and countries within the European Union [2]. Polymyxin B has been used as a clinical antibiotic for more than half a century [3]. Many AMPs have also been investigated for practical use [4]. Microbial killing by AMPs is often correlated mainly with membrane disruption although some other intracelluar and extracellular mechanisms also contribute to overall activity [1]. Several AMPs such as indolicidin attack intracellular targets without membrane disruption [5].

Using combinations of agents is common in a clinical setting in order to obtain more effective antimicrobial properties. Such combinatorial application is also effective for AMPs. Conventional low-molecular-mass antimicrobials often exhibit synergistic effects with AMPs [6]. Synergy is also observed in some combinations of AMPs naturally coexisting in the tissues of producing organisms, e.g., magainin 2 and PGLa [7], different isomers of dermaseptins and temporins [8,9], cathelicidins and defensins [10], β-defensin and BPI [11], hepcidin and moronecidin [12], Cg-Prp and Cg-Def [13], and AFP and sarcotoxin IA [14]. Certain artificial combinations of AMPs isolated from distinct organisms are synergistic, e.g., some eukaryotic AMPs and bacteriocins [15], and magainin and tachyplesin I [16]. Lysozymes, 1,4-β-*N*-acetylmurmidases with membrane-perturbing activity, are synergistic with many AMPs [17,18]. The staphylococcal glycylglycine endopeptidase lysostaphin is also synergistic with polymyxin B and ranalexin [19,20]. All synergies mentioned above are found in combinations of AMPs and other antimicrobials including AMPs.

**Figure 1:**
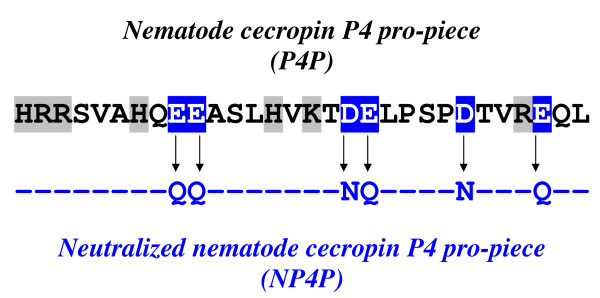
**Structure of NP4P**. The parent peptide, nematode cecropin P4 pro-piece (P4P), is shown at the top. Inversed letters indicate acidic amino acid residues which were substituted with amides in NP4P. Letters on a grey background represent basic amino acid residues.

Here, we describe potent enhancement of AMP activities by a synthetic peptide NP4P (Y. Kato, K. Kusaka, S. Ueno, H. Zhang, and M. Minaba, 8 May 2008, Japanese Patent Office). Increase in positive charge facilitates the interaction of peptides with negatively charged biological membranes, and often results in the conferring of membrane-disrupting or membrane-penetrating activities. We generated some peptides derived from natural non-antimicrobial sequences, with modification to confer a cationic net charge. These peptides were then subjected to screening for novel AMPs that have structures distinct from those of known AMPs. NP4P was originally one of these peptides. The parent peptide of NP4P was a non-antimicrobial peptide fragment, nematode cecropin P4 pro-region (P4P, calculated pI = 5.80) [21,22]. NP4P was generated from P4P by substitution of all acidic amino acid residues with amides (i.e., Glu → Gln, and Asp → Asn), resulting in a reduction of negative charge and an acquisition of stronger net positive charge (Figure 1). It consisted of 30 amino acid residues and was highly basic (calculated pI = 12.30). When evaluating the pharmacological properties of NP4P, we found that NP4P enhanced the activities of some AMPs whereas no antimicrobial activity was detected for NP4P alone, suggesting that the effect of NP4P was an enhancement, but not a synergy as mentioned above. This study is the first report on the unique features of NP4P.

## Results and Discussion

### Evaluation of antimicrobial activity of NP4P

**Figure 2:**
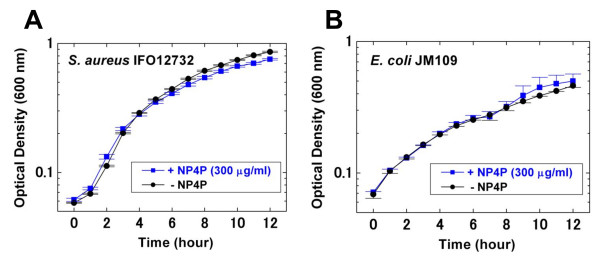
**Effect of NP4P on bacterial growth**. *Staphylococcus aureus *IFO12732 (A) and *Escherichia coli JM109 *(B) in the logarithmic phase were suspended in 2 mL of IFO702 medium with or without 300 μg/mL of NP4P. Their optical densities were adjusted to 0.06-0.08 at 600 nm. The bacterial suspension was incubated at 30°C. Bacterial growth was estimated by measuring the change in optical density. All experiments were performed in triplicate. Each data point represents the mean ± SEM.

Antimicrobial activity was evaluated as the first step in pharmacological characterization of NP4P. We did not detect antimicrobial activity for NP4P at ≤ 300 μg/mL in growth-inhibitory and microbicidal assay against certain Gram-positive bacteria (*Staphylococcus aureus *IFO12732, *Bacillus subtilis *IFO03134, and *Micrococcus luteus *IFO 12708); Gram-negative bacteria (*Pseudomonas aeruginosa *IFO3899, *Salmonella typhimurium *IFO13245, *Serratia marcescens *IFO3736 and *Escherichia coli *JM109); and yeast (*Saccharomyces cerevisiae *MAFF113011), as well as against monkey Vero kidney cells (data not shown). In addition, NP4P (300 μg/mL) did not affect the growth curves of *S. aureus *IFO12732 (Figure 2A) and *E. coli *JM109 (Figure 2B). These results indicate that NP4P was less toxic to microbes.

### Enhancer activity for antimicrobial peptides

**Figure 3:**
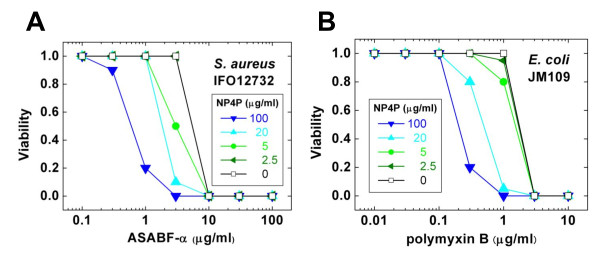
**NP4P enhancement of bactericidal activities of AMPs**. The dose-effect curves were determined in the presence of NP4P at various concentrations (0, 2.5, 5, 20, and 100 μg/mL). Bactericidal activities were measured against *S. aureus *IFO12732 for ASABF-α (A) and against *E. coli* JM109 to polymyxin B (B). Viability is defined as normalized number of viable cells to the number in the absence of ASABF-α or polymyxin B.

The parent peptide P4P inhibited the bactericidal activity of cecropin P4 and some other antimicrobial peptides ([22]; S. Ueno and Y. Kato, unpublished data), encouraging us to test whether NP4P affected the activities of other antimicrobial agents. We examined the effect of NP4P on the bactericidal activities of the nematode CSαβ-type cationic AMP ASABF-α [23-25] against *S. aureus *IFO12732 (Figure 3A) and polymyxin B against *E. coli *JM109 in 10 mM Tris-HCl, pH 7.4 (Figure 3B). Unexpectedly, NP4P enhanced these activities at ≥ 5 μg/mL in a dose-dependent manner. The dose-effect curves of ASABF-α and polymyxin B were shifted to almost 10 times lower concentration in the presence of 100 μg/mL NP4P. However, the enhancement was completely abolished in a high ionic strength condition (150 mM NaCl, 50 mM NaHCO_3_, 10 mM Tris-HCl, pH 7.4).

Furthermore, we tested NP4P enhancement at 20 μg/mL for the activities of antimicrobial agents against various microbes (Table 1). The results can be summarized as: (1) The bactericidal activities of all tested membrane-disrupting AMPs (ASABF-α, polymyxin B, and nisin) were enhanced. (2) The enhancement was selective depending on the type of bacterial species. For instance, the activities of ASABF-α against *S. aureus *IFO12732 and *E. coli *JM109 were enhanced, whereas a lesser enhancement was observed against *M. luteus *IFO 12708, *B. subtilis *IFO3134, *P. aeruginosa *IFO3899, and *S. marcescens *IFO3736. (3) NP4P did not enhance the activity of one AMP indolicidin which killed bacteria by inhibition of DNA synthesis and not by membrane disruption [5]. (4) NP4P did not affect the activities of conventional antimicrobial agents that do not target bacterial cytoplasmic membranes (ampicillin, kanamycin, and enrofloxacin).

**Table 1:** Effect on MBC values of various antimicrobial agents

	MBC (μg/mL)	
	NP4P-^a^	NP4P+
ASABF-α^b^		
*Staphylococcus aureus *IFO12732	3	0.3
*Micrococcus luteus *IFO12708	5	2
*Bacillus subtilis*IFO3134	8	3
*Escherichia coli *JM109	3	0.3
*Pseudomonas aeruginosa *IFO3899	5	2
*Salmonella typhimurium *IFO13245	3	2
*Serratia marcescens *IFO3736	3	1.5
Polymyxin B^b^		
*Escherichia coli *JM109	3	0.3
*Pseudomonas aeruginosa *IFO3899	5	2.5
*Salmonella typhimurium *IFO13245	5	2.5
*Serratia marcescens *IFO3736	5	1
Nisin^b^		
*Staphylococcus aureus *IFO12732	5	2
Indolicidin^c^		
*Staphylococcus aureus *IFO12732	10	10
*Escherichia coli *JM109	10	10
Ampicillin^c^		
*Staphylococcus aureus *IFO12732	250	250
Kanamycin^c^		
*Staphylococcus aureus *IFO12732	3	3
Enrofloxacin^c^		
*Staphylococcus aureus *IFO12732	0.25	0.25

^a ^Each MBC value was determined in the presence or absence of 20 μg/mL NP4P.

^b ^Membrane disruptive.

^c ^Not membrane disruptive.

### Effect on disruption of the cytoplasmic membrane

**Figure 4:**
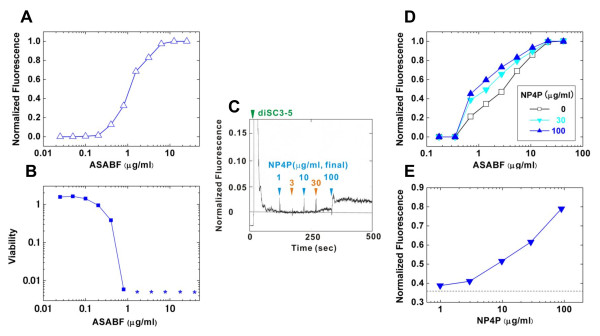
**Effect of NP4P on the membrane-disrupting activity of ASABF-α against the cytoplasmic membrane of *S. aureus***. Disruption of the cytoplasmic membrane was estimated by the increase in fluorescence intensity of diS-C_3_-(5). Changes in fluorescence were normalized by the value at the plateau of the dose-response curves. (A) Dose-response curve and (B) dose-bactericidal effect curve of ASABF-α against *S. aureus *IFO12732. These curves were simultaneously determined. The asterisks indicate that viable cells were not detected. (C) Effect of NP4P on the cytoplasmic membrane. The time courses of fluorescence changes are represented. (D) Effect of NP4P on cytoplasmic membrane disruption by ASABF-α. Dose-response curves were determined in the presence of NP4P at various concentrations (0, 30, and 100 μg/ml). (E) Another assay for NP4P enhancement. NP4P was applied after treatment of 1.28 μg/mL of ASABF-α. The fluorescent change evoked only by ASABF-α is indicated by a dashed line.

NP4P enhancement was observed only for the antimicrobial activities of membrane-disrupting AMPs. The simplest hypothesis accounting for NP4P enhancement was direct facilitation of membrane disruption. To test this hypothesis, we examined the effect of NP4P on the activity of bacterial membrane disruption by ASABF-α. diS-C_3_-(5) is a slow-response voltage-sensitive fluorescent dye [26]. The extracellularly administered diS-C_3_-(5) accumulates on the hyperpolarized cell membrane, translocates into the lipid bilayer, and redistributes between the cells and the medium in accordance with the membrane potential. Aggregation within the confined membrane interior or intracellular spaces usually results in reduced fluorescence by self-quenching. Depolarization or disruption of the cytoplasmic membrane causes the release of diS-C_3_-(5) from the cells to the medium and an increase in fluorescence intensity. ASABF-α evoked the increase in fluorescence against diS-C_3_-(5)-loaded *S. aureus *IFO12732 in a dose-dependent manner (Figure 4A). ASABF-α induced calcein (molar mass = 622.53) leakage from the acidic-liposomes (data not shown), indicating that the increase in fluorescence was attributed to leakage of diS-C_3_-(5) by membrane disruption rather than redistribution by depolarization. Bactercidal activity was parallel to the release of diS-C_3_-(5) (Figure 4B), suggesting that ASABF-α killed *S. aureus *mainly by disruption of the cytoplasmic membrane.

The effect of NP4P was investigated using this experimental setting. NP4P evoked no significant change in fluorescence at ≤ 10 μg/mL whereas weak ripples or limited increase were observed at higher concentrations (2.5% of maximal response at 100 μg/mL: the maximal response was defined as the increase in fluorescence at the plateau in the dose-response curve of ASABF-α) (Figure 4C). In addition, NP4P did not disrupt the acidic-liposomal membrane at ≤ 220 μg/mL (data not shown). This suggests that NP4P barely affected either the membrane permeability or membrane potential of *S. aureus*. To test the effect of NP4P on the membrane-disrupting activity of ASABF-α, dose-response curves were determined in the presence or absence of NP4P (Figure 4D). The efficacy of membrane disruption by ASABF-α was remarkably enhanced by NP4P in a dose-dependent manner. The threshold concentration of ASABF-α was not significantly affected. Several doses of NP4P were added to *S. aureus *which was intermediately damaged by 1.28 μg/mL of ASABF-α [36% increase in maximal response in diS-C_3_-(5) fluorescence] (Figure 4E). Even 1 μg/mL of NP4P caused detectable enhancement. The degree of enhancement increased dose-dependently. These results suggest that NP4P enhances the bactericidal activity of ASABF-α by increasing the efficacy of membrane disruption.

AMPs from the skin of a frog, PGLa and magainin 2, form heterodimers and show synergistic membrane disruption and antimicrobial activities [7,27]. NP4P is not as likely to bind directly with AMPs as PGLa and magainin 2 because the structure of ASABF-α, nisin, and polymyxin B, whose bactericidal activities were enhanced by NP4P, are completely distinct [28-30]. NP4P is a highly basic molecule and could interact with negatively charged cytoplasmic membranes. A possible mechanism of NP4P enhancement is destabilization of the cytoplasmic membrane. Whereas NP4P did not exhibit neither growth inhibitory nor bactericidal activity against *S. aureus *at ≤ 200 μg/ml, ripples or weak increase in diS-C_3_-(5) fluorescence was evoked at > 10 μg/mL, suggesting that NP4P interacted with bacterial cytoplasmic membranes and caused sublethal membrane destabilization. For acidic liposomes, NP4P neither evoked such membrane destabilization-like responses nor enhanced membrane disruption of ASABF-α. However, the results obtained by quantifying bacterial membrane disruption using using diS-C_3_-(5) may indicate the more specific mode of action. The intensities of enhancement did not correlate with the susceptibilities of the bacteria for the tested AMPs. The killing of *E. coli *JM109 was most efficiently enhanced for ASABF-α and polymyxin B, suggesting that the efficacy of NP4P enhancement depends on the species of bacteria rather than on that of AMPs. These results support our hypothesis that NP4P independently interacts with cytoplasmic membranes and not with AMPs. For acidic liposomes, membrane disruption of ASABF-α was inhibited in the presence of 20 μg/mL NP4P. The dose-response curve was shifted to a higher concentration (IC_50 _= 0.23 μg/mL without NP4P, and 0.53 μg/ml with NP4P), indicating that NP4P was a competitive inhibitor. This inhibition could be due to charge neutralization of the membrane surface by NP4P binding and prevention of ASABF-α binding in a similar manner to that observed between magainin 2 and an acyclic tachyplesin I analogue [16], i.e., NP4P and ASABF-α also bind to the liposomal membrane independently. This observation does not contradict our hypothesis mentioned above. The exact mechanisms for NP4P enhancement at the molecular level remains to be elucidated.

## Conclusions

NP4P selectively enhances the bactericidal activities of membrane-disrupting AMPs (ASABF-α, nisin, and polymyxin B). NP4P is not bactericidal and does not inhibit growth at ≤ 300 μg/mL against all tested bacteria, suggesting that the effect of NP4P is enhancement and is distinct from the previously reported synergy among AMPs and/or low-molecular mass antimicrobials [6-20]. Enhancement intensities depend on microbial species. Relatively good enhancement was achieved for *S. aureus *and *E. coli*. Increasing the efficacy of membrane disruption against the bacterial cytoplasmic membrane may contribute to enhancement by NP4P.

AMPs are immune effectors against microbial infections in vertebrates, invertebrates, and plants. In humans, the deficiency in AMP functions often causes reduced resistance against infectious diseases [31,32], indicating that resistance may increase by enhancing the effect of AMPs. AMP-enhancers without antimicrobial activities are promising as immunopotentiators since they do not disturb the autonomic control of immunity. Although salt-inhibition remains to be resolved for practical use in mammals, NP4P is believed to be the first peptide which exerts AMP-enhancer activity.

## Methods

### Microorganisms

*E. coli *JM109 was purchased from Takara (Otsu, Japan). Other strains described below were transferred from the National Institute of Technology and Evaluation, Kazusa, Japan: *S. aureus *IFO12732, *B. subtilis *IFO3134, *M. luteus *IFO12708, *P. aeruginosa *IFO3899, *S. typhimurium *IFO13245 and *S. marcescens *IFO3736.

### Peptides and other AMPs

NP4P, cecropin P4 and indolicidin were prepared at Biologica Co. (Nagoya, Japan). Briefly, peptides were synthesized by the Fmoc method, and purified by reversed-phase high-pressure liquid chromatography. The products were confirmed by time-of-flight mass spectrometry on a Voyager DE Mass Spectrometer, Applied Biosystems (Foster City, CA, USA). ASABF-α was prepared as previously described [24]. Some antimicrobials were purchased from Wako, Osaka, Japan (ampicillin, kanamycin, and polymyxin B); Sigma, St. Louis, MO, USA (nisin); and Bayer, Nordrhein-Westfalen, Germany (enrofloxacin).

### Growth assay

Microbes in the mid-exponential phase were suspended in 2 mL of IFO702 medium (1% polypeptone, 0.2% yeast extract, 0.1% MgSO_4_/7H_2_O) with or without NP4P. Their optical densities were adjusted to an OD_600 _of 0.06-0.08. The bacterial suspension was incubated at 30°C. Bacterial growth was estimated by measuring the change in OD_600_.

Monkey Vero cells were grown in 2 ml of Dulbecco's modified Eagle's medium supplemented with 5% fetal bovine serum at 37°C and 5% CO_2_. To estimate cytotoxicity, NP4P was added to the medium at 0, 30, 100, and 300 μg/mL. Cell proliferation and morphorogy were monitored for a week.

### Microbicidal assay

Microbicidal assay was performed as previously described [33]. Briefly, each microbial strain in the mid-exponential phase was suspended in 10 mM Tris/HCl, pH 7.5. The microbial suspension was mixed with antimicrobials in the presence or absense of NP4P. After 2 h incubation, the suspension was diluted 1,000 times and inoculated on to plates of IFO702 medium. The number of colonies were counted, and a plot of peptide concentration vs colony number was created.

### Liposome disruption assay

Membrane-disrupting activity was estimated by liposome disruption assay [33]. A lipid film was prepared by rotary evaporation of lipid solution [1 mg lipid in 1 mL chloroform, phosphatidylglycerol (mole):caldiolipin (mole) = 3:1]. The lipid film was hydrated with 1 mL of 10 mM Tris-HCl buffer (pH 7.5) containing 75 mM calcein. Lipid dispersions were sonicated and subjected to five freeze-thaw cycles. Non-trapped calcein was removed by gel filtration on a Sephacryl S-300 spin column (GE Healthcare Bio-Science Corp., Piscataway, NJ, USA) equilibrated with 10 mM Tris-HCl (pH 7.5) containing 175 mM NaCl and 1 mM EDTA. These calcein-entrapped liposomes were diluted at a ratio of 1:1000 in 10 mM Tris-HCl (pH 7.5) containing 350 mM sucrose. Calcein release after membrane disruption was evaluated by measuring fluorescence intensity at 515 nm with excitation at 492 nm on a Shimadzu RF-5300PC spectrofluorometer (Shimadzu, Kyoto, Japan) at room temperature.

### Cytoplasmic membrane permeability assay

Cytoplasmic membrane permeabilization of *S. aureus *was determined with a voltage-sensitive dye, diS-C_3_-(5) [34,35]. Bacteria in the mid-exponential phase were suspended in 10 mM Tris-HCl with or without NP4P, pH 7.5 to an OD_600 _of 0.05. Changes in fluorescence were continuously monitored using a Shimadzu RF-5300PC spectrofluorometer at an excitation wavelength of 622 nm and an emission wavelength of 670 nm. The bacterial suspension was incubated with 400 nM diS-C_3_-(5). ASABF-α was added to the bacterial suspension after the dye uptake was maximal. The maximal increase in fluorescence due to disruption of the cytoplasmic membrane was recorded.

## Authors' contributions

SU, KK, and YK designed and performed most of the experimental work. SU and YT performed the experiment using liposomes. MM and HZ has mainly performed the antimicrobial assay. YK edited the manuscript. This study conducted completely under the supervision of YK. All authors read and approved the final manuscript.
